# Cross-specific imprinting tells the seed size of hybrids

**DOI:** 10.1093/plphys/kiae154

**Published:** 2024-03-13

**Authors:** Dechang Cao

**Affiliations:** Assistant Features Editor, Plant Physiology, American Society of Plant Biologists; Germplasm Bank of Wild Species, Kunming Institute of Botany, Chinese Academy of Sciences, Kunming, Yunnan 650201, China

Mules are widely used in farms over the world and might be the earliest and most successful use of “hybrid vigor” by human beings. This expression reflects that in many cases, hybrids show superior growth and fitness over their parents. This phenomenon is also termed as “heterosis” in agriculture, and taking advantage of heterosis in plant and animal breeding is a landmark of modern agriculture (Shull 1908). The regulatory mechanisms underlying heterosis have been extensively studied but remain elusive (Chen 2013), albeit heterosis has been widely used in breeding of many globally important crops, including rice, maize, and sorghum (Paril et al. 2024).

Heterosis shows parent-of-origin effects; that is, the superior performance of hybrid offspring is dependent on maternal and paternal contributions in the reciprocal crosses. For example, a female horse bears mules while a female donkey bears hinnies in the reciprocal crosses between horses and donkeys. The best characterized mechanism underlying parent-of-origin effects is genomic imprinting (Lawson et al. 2013). Imprinting is a phenomenon mediated by epigenetic modifications that causes unequal expression of the maternal and paternal alleles of a gene. However, cytoplasmic-nuclear interactions also lead to phenotypic variation of the hybrid offspring (Tang et al. 2013). Thus, the effects of cytonuclear interactions cannot be simply ruled out in the parent-of-origin effect of heterosis. In this issue of *Plant Physiology*, June et al. (2024) dissected these confounding effects on seed size heterosis of Arabidopsis (*Arabidopsis thaliana*) hybrids.

Application of cytoplasmic-nuclear substitution (CNS) lines in reciprocal crosses provided an elaborate solution to dissect the effects of imprinting and cytonuclear interactions. CNS lines can be established by backcrossing with the recurrent paternal parent for 5 to 6 generations such that the cytoplasmic genome will be retained while the nuclear genome is replaced (Fig. A), essentially creating a cytoplasmic hybrid (cybrid, i.e. the cytoplasm combined with a different nucleus). June et al. (2024) established such CNS lines of Arabidopsis using the ecotypes of Col-0 and C24. By reciprocal crosses of the CNS lines, they found that the hybrids showed similar seed size when the crossed lines have the same nuclear genome and different cytoplasmic genomes, that is, no cytonuclear effect (June et al. 2024). By contrast, in the reciprocal cross of CNS lines with the same cytoplasmic genome but different nuclear genomes, the seed size differed by ∼20%, meaning the imprinting effect was detected. Thus, imprinting alone determines the seed size heterosis in these CNS hybrids (Fig. B).

**Figure.**
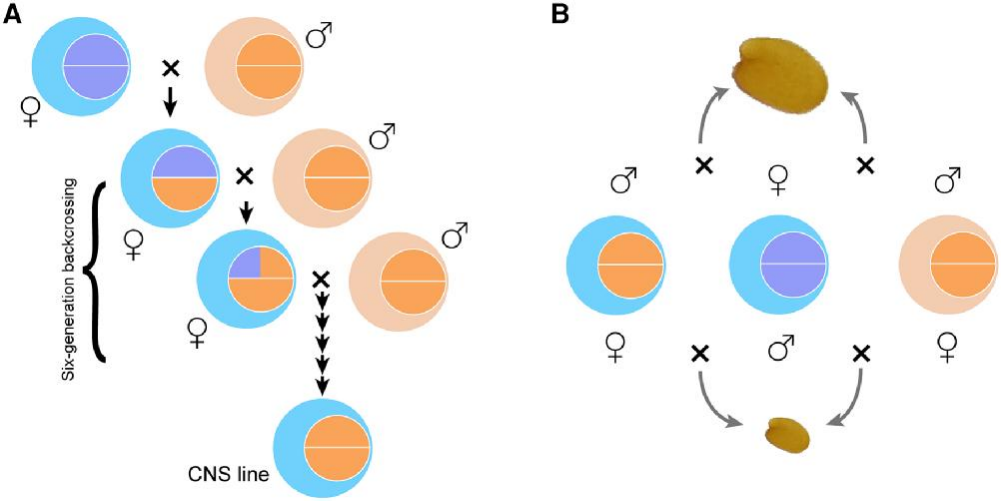
Imprinting rather than cytonuclear interactions determines seed size heterosis in Arabidopsis hybrids. **A)** A scheme showing generation of the CNS lines via several generations of backcrossing. The larger circles represent the cytoplasm, and the smaller circles represent the genetic contribution of nuclear genome. After 6 generations of backcrossing with the recurrent paternal parent, the CNS lines are generated with the nuclear genome replaced while the cytoplasm retained. **B)** Parent-of-origin effects on seed size heterosis in Arabidopsis. The CNS line resembles the paternal parent to determine hybrid seed size in the reciprocal crosses.

The seed size difference in the CNS hybrids was observed as early as 7 days after pollination (DAP). June et al. (2024) isolated early-stage embryos and endosperms at 6 DAP by laser capture microdissection to analyze differentially expressed genes, including imprinted genes associated with seed size heterosis. A total of 99 maternally expressed genes (MEGs) and 89 paternally expressed genes (PEGs) were characterized in the endosperm of the reciprocal crosses, with a few additional MEGs and PEGs found in the reciprocal cybrid hybrids (different cytoplasms with the same nuclei) and the conventional reciprocal crosses. Compared to a survey into previously reported imprinting genes, around two-thirds of known MEGs and one-half of known PEGs were shared among the CNS and conventional hybrids.

Cross-specific imprinted genes (with biased expression in one of the reciprocal crosses) have been reported (Pignatta et al. 2014) and might contribute to the parent-of-origin effect on heterosis. Indeed, June et al. (2024) found 11 cross-specific imprinting genes in the CNS crosses. They further tested if these cross-specific imprinted genes are associated with DNA methylation using published data from the 1,001 epigenomes project (Kawakatsu et al. 2016).

One gene encoding the homeodomain glabrous 9 (*HDG9*) transcriptional factor displayed an allelic methylation pattern. Methylation showed varying patterns at the 5′ region of *HDG9* among the different genotypes of Col, Cvi, and L*er*, with a total loss of 5′ methylation in the genotype C24. Imprinting of *HDG9* was detected in crosses where the paternal allele is methylated and the maternal allele is not, and the imprinting was lost in the reciprocal crosses. It is likely that the changes in imprinting of *HDG9* could be associated with the seed size heterosis (June et al. 2024), so to test this hypothesis, knock-out (KO) lines were generated in C24 and Col-0 backgrounds using CRISPR/Cas9. KO lines of *HDG9* in C24 indeed showed increased seed size, while no significant effect was observed in *HDG9* KO lines in Col-0. These results suggest that cross-specific imprinting of *HDG9* may contribute to parent-of-origin seed size heterosis.

The considerable number of cross-specific imprinted genes found in the endosperm suggests an essential role of maternal imprinting in seed size heterosis. RNA-directed DNA methylation (RdDM) has been well characterized as a pathway regulating maternal imprinting (Cao 2023; Dew-Budd et al. 2023). RdDM is triggered by 24-nt small interfering RNAs generated from the RNA Pol IV transcripts (Xie et al. 2024). June et al. (2024) observed varied seed sizes between reciprocal crosses between C24 and mutants deficient in Pol IV function. Thus, the maternal instead of the paternal *NRPD1* allele should be responsible for the seed size heterosis.

Taken together, the study by June et al. (2024) provided interesting evidence that imprinting and cross-specific imprinting in particular plays an essential role in seed size heterosis. However, not all results are straightforward, and some do not give positive outcome. For example, the long noncoding RNA encoded by *AT4G13945* was paternally imprinted in the embryo but did not show any embryo or seed size phenotypes in the knockout lines. It is possible that heterosis is a trait-specific and species-dependent phenomenon, and the seed size is largely dependent on the endosperm instead of embryo development. The essential roles of maternal imprinting in parent-of-origin seed size heterosis could provide valuable clues for breeding future crops with superior seed yield.

## Dive Curated Terms

The following phenotypic, genotypic, and functional terms are of significance to the work described in this paper:

HDG9 Gramene: AT5G17320
HDG9 Araport: AT5G17320

heterosis Planteome: TO:0000355

NRPD1 Gramene: AT1G63020
NRPD1 Araport: AT1G63020
